# Screening and transcriptomic analysis of the ethanol-tolerant mutant *Saccharomyces cerevisiae* YN81 for high-gravity brewing

**DOI:** 10.3389/fmicb.2022.976321

**Published:** 2022-08-25

**Authors:** Tianyou Yang, Shishuang Zhang, Linbo Li, Jing Tian, Xu Li, Yuru Pan

**Affiliations:** School of Life Sciences and Technology, Henan Institute of Science and Technology, Xinxiang, China

**Keywords:** craft beer, *Saccharomyces cerevisiae*, ethanol tolerance, trehalose, transcriptomic, high-gravity brewing

## Abstract

Ethanol stress is one of the major limiting factors for high-gravity brewing. Breeding of yeast strain with high ethanol tolerance, and revealing the ethanol tolerance mechanism of *Saccharomyces cerevisiae* is of great significance to the production of high-gravity beer. In this study, the mutant YN81 was obtained by ultraviolet-diethyl sulfate (UV-DES) cooperative mutagenesis from parental strain CS31 used in high-gravity craft beer brewing. The ethanol tolerance experiment results showed that cell growth and viability of YN81 were significantly greater than that of CS31 under ethanol stress. The ethanol tolerance mechanisms of YN81 were studied through observation of cell morphology, intracellular trehalose content, and transcriptomic analysis. Results from scanning electron microscope (SEM) showed alcohol toxicity caused significant changes in the cell morphology of CS31, while the cell morphology of YN81 changed slightly, indicating the cell morphology of CS31 got worse (the formation of hole and cell wrinkle). In addition, compared with ethanol-free stress, the trehalose content of YN81 and CS31 increased dramatically under ethanol stress, but there was no significant difference between YN81 and CS31, whether with or without ethanol stress. GO functional annotation analysis showed that under alcohol stress, the number of membrane-associated genes in YN81 was higher than that without alcohol stress, as well as CS31, while membrane-associated genes in YN81 were expressed more than CS31 under alcohol stress. KEGG functional enrichment analysis showed unsaturated fatty acid synthesis pathways and amino acid metabolic pathways were involved in ethanol tolerance of YN81. The mutant YN81 and its ethanol tolerance mechanism provide an optimal strain and theoretical basis for high-gravity craft beer brewing.

## Graphical Abstract

Table of contents graphics (TOC). 
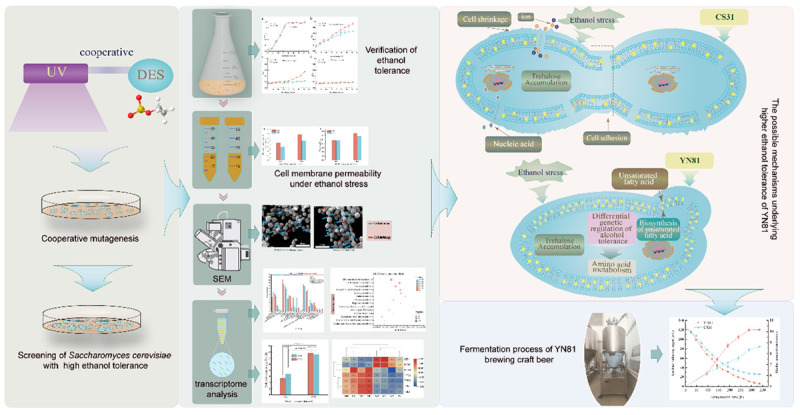

## Introduction

In industrial breweries, the majority of beer currently produced is lager, which is brewed to satisfy commercial tastes rather than more determined palates. Craft breweries typically have a small capacity but can brew different varieties of beer depending on the malt, hops, and *Saccharomyces cerevisiae*, making them more enticing to the average taste-and-aroma-conscious consumer. In recent decades, there has been a notable change in the behavior of a portion of beer consumers, who pay more attention to craft beer (Aquilani et al., 2015; Li T. et al., 2020). And, unlike large-scale industrial beer, craft beer, is produced with more concern for quality and diversity based on preserving original sensory characteristics (Hayward et al., 2019). High-alcohol beers are becoming increasingly popular with consumers (Jaeger et al., 2020), especially the Chinese who prefer high-alcohol beverages (Zhang et al., 2022).

The high-alcohol beer is commonly brewed with high-gravity worts (Silva et al., 2008; Puligundla et al., 2011). Nonetheless, the high-alcohol beer brewing with high-gravity worts imposes challenges for sequential repitching due to the osmotic stress that *S. cerevisiae* cells experience in the principal long stretches of fermentation, which are followed by ethanol, nutritional, and cold stress in the later phases of fermentation (Gibson et al., 2007; Nikulin et al., 2018). The stress conditions can result in *S. cerevisiae* with low fermentability, preventing them from being used in subsequent fermentations (Huuskonen et al., 2010). Ethanol toxicity to *S. cerevisiae* is a primary stressor for high-alcohol beer brewing with high-gravity worts (Stanley et al., 2010).

In spite of the fact that *S. cerevisiae* has evolved to tolerate ethanol with certain levels, it still is negatively affected when ethanol accumulates to a high concentration during fermentation. Moderate concentrations of ethanol (around 5% v/v) can alter the enzymatic kinetics of proteins associated with primary metabolism (e.g., glycolysis), and affect different biological processes. In comparison, high concentrations of ethanol (>5% v/v) can cause yeast cell damage, involved in increasing cell membrane permeability, decreasing cell viability, inhibiting cell growth, and changing cell morphology (Yang et al., 2018b; Telini et al., 2020). Actually, it has been reported that tolerance of *S. cerevisiae* to ethanol toxicity is related to intracellular trehalose content, the fluidity and composition of cell membrane as well as the levels of unsaturated fatty acids, ergosterol, amino acids, inositol, heat shock proteins (HSPs), ATPase and storage carbohydrates (Samakkarn et al., 2021). Hence, the low ethanol tolerance of *S. cerevisiae* is becoming a bottleneck for high-gravity brewing. However, current commercial *S. cerevisiae* has low ethanol tolerance, so obtaining a yeast strain with high-ethanol tolerance is an important approach to achieve high-gravity brewing. In past decades, researchers used various mutagenesis methods to screen high-ethanol tolerance yeast, such as mutagenesis (Yi et al., 2018), adaptive evolution (Ekberg et al., 2013), protoplast fusion (Xin et al., 2020), and genetic engineering (Jetti et al., 2019). Physical and chemical cooperative mutagenesis is one of the most commonly used mutation techniques, which is characterized by simple operation, high efficiency, high mutation rates, and ease of dissemination. For example, Du et al. (2017) obtained a mutant strain A9-2 by UV-DES cooperative mutagenesis, and the fermentability and ethanol production ability of this strain was significantly higher than that of the parental strain A9.

Transcriptomic analysis is a powerful tool for the study of the global gene expression dynamics of *S. cerevisiae* in response to various environmental stresses (Mardanov et al., 2020). Moreover, RNA sequencing (RNA-seq) has already been successfully applied to *S. cerevisiae* (Nagalakshmi et al., 2008), which can improve our knowledge of the complexity of eukaryotic transcriptomes. Various genes regulate the phenotypic characteristics of *S. cerevisiae*, and the expression of different genes involved in multiple biological functions and processes has been found to be remarkably altered in response to ethanol stress (Li et al., 2017). Previous transcriptomic analysis showed that ethanol stress could induce the active expression of membrane-related genes (Avrahami-Moyal et al., 2012), unsaturated fatty acid synthesis (Samakkarn et al., 2021), amino -acid-metabolism-related genes (Wang et al., 2021), and trehalose-related genes.

In this study, *S. cerevisiae* CS31 was used as the parental strain. We generated the mutants with UV-DES mutagenesis, and then selected strains with higher ethanol based on growth in ethanol-containing media. The changes in cell growth, cell viability, cell membrane permeability, and trehalose content of the mutant strain YN81 were investigated, compared with those of CS31. More crucially, the tolerance mechanism of the mutant strain YN81 was revealed through transcriptomic analysis.

## Materials and methods

### Materials

*S. cerevisiae* CS31 (parental strain, purchased from Angel Yeast Co., Ltd.); YPD medium (glucose 20 g/L, peptone 20 g/L, yeast extract 10 g/L). YPD-ethanol screening medium (YPD medium with 9% v/v, ethanol).

### Ultraviolet-diethyl sulfate cooperative mutagenesis

To obtain strain with high-ethanol tolerance, the cell suspension of *S. cerevisiae* CS31 was treated with ultraviolet (UV) and diethyl sulfate (DES). For UV mutagenesis, CS31 cell suspension (10^6^ CFU/mL) was irradiated at a distance of 35 cm from UV light (35 W) for 0, 40, 80, 120, 160, 200, and 240 s. After irradiation, the suspension was diluted appropriately and spread on YPD agar plates, and incubated at 28^°^C in darkness for 36 h. For DES treatment, the suspension was treated with 0.05% (v/v) DES for 0, 15, 35, 45, 60, and 75 min and in five concentrations [0, 0.05, 0.1, 0.15, and 0.2% (v/v)] for 30 min, respectively, and finally 25% sodium thiosulfate was used to terminate the procedure. Treated cells were diluted with sterile water, and then spread on YPD agar plates and incubated at 28^°^C for 36 h. The optimal mutagenesis condition was determined by the lethality of about 70–80% (Yi et al., 2018). Cooperative mutagenesis of CS31 was carried out after determining the optimal mutagenic parameters of UV and DES.

Lethality was calculated as follows:


$$\text{Lethality}\left(\%\right)=\frac{\begin{array}{c} \text{Number of strains before mutation}\\ -\text{ Number of strains}\,\mathrm{after}\,\mathrm{mutation} \end{array}}{\text{Number of strains}\,\mathrm{before}\,\mathrm{mutation}}\times 100$$

### Primary screening and rescreening of mutants

After mutagenesis, cells were collected and washed 3 times with phosphate buffered solution (PBS), and then, diluted cell suspensions were spread on YPD-alcohol screening medium and cultured at 28°C. The colonies with a diameter larger than 10% of the parental strain were selected and transferred to YPD medium for incubation. The preliminary screening strain was inoculated into 100 mL of YPD liquid medium containing 9% ethanol and cultured at 28°C for 24 h. The rescreening strain was selected according to the cell viability detected by methylene blue staining (Zhao et al., 2014).

### Analysis on ethanol tolerance of mutant

Cell suspensions of mutants and the parental strain CS31 were cultured in YPD medium containing 0, 9, 10, and 11% ethanol at 28°C. The inoculation amount was 3% (v/v). The cell concentration was measured by OD_600_, and cell viability was measured by methylene blue staining.

### Cell morphology assessment by scanning electron microscope

Pretreatment of cell samples was performed according to Yang et al. (2018b). *S. cerevisiae* was cultured at 28°C and 120 rpm for 36 h in YPD medium containing 10% ethanol. These cells were harvested by centrifugation, rinsed with PBS, and cells were fixed with 2.5% (v/v) glutaraldehyde solution for 2 h at 4°C and then rinsed with PBS for three times. Rinsed cells were dehydrated in a series of ethanol concentrations (30, 50, 60, 70, 90, 95, and 100%) and dried to the critical point (Samdri-PVT-3D). The cells were coated with gold (MSP-2S) and examined using a scanning electron microscope (SEM) (Quanta 200, America).

### Cell membrane permeability

Relative electrical conductivity (REC) of cells and extravasation of intracellular nucleic acid as indicators for evaluating the permeability of cell membranes under ethanol stress. The determination of the REC of the cells was performed according to Yi (2015). *S. cerevisiae* cells were centrifuged, rinsed with sterile water three times, and then resuspended in the same volume of sterile water. Following that, the conductivity of the cells was measured by Conductometer (DDS-307, China), and the REC was calculated using the equation below.


$$\mathrm{Relative}\,\mathrm{electrical}\,\mathrm{conductivity}=\,\frac{C_{1}-C_{0}}{C_{2}-C_{0}^{\prime }}\times 100\%$$

*C*_1_ and *C*_0_: the electrical conductivity of *S. cerevisiae* suspension and sterile water, respectively.

*C*_2_ and *C*_0_′: the electrical conductivity of *S. cerevisiae* suspension and sterile water after boiling for 5 min, respectively.

The determination of nucleic acid content was performed according to Liu et al. (2004). To eliminate the extracellular enzymes, 5 mL of *S. cerevisiae* suspension was incubated at 80°C for 5 min. Then, the *S. cerevisiae* suspension was centrifuged at 5,000 g for 20 min, and 0.2 mL of the supernatant was diluted 200 times to determine the absorbance at 260 nm.

### Intracellular trehalose concentration

*S. cerevisiae* cells were harvested by centrifugation and washed with sterile water. To extract trehalose from the cells, an equal volume of 0.5 mol/L trichloroacetic acid solution was added and sonicated for 45 min. 60 μL of the intracellular extract was incubated with 240 μL of an 80% sulfuric acid solution containing 0.2% anthrone for 5 min in a boiling water bath. The trehalose concentration was measured by the absorbance at 620 nm and compared to samples containing known trehalose concentrations (Zhang et al., 2019). Standard curves were drawn based on the absorbance values of various concentrations of trehalose.

### Transcriptomic analysis

The cells were harvested by centrifugation, washed with sterile normal saline, frozen in liquid nitrogen, and stored at –80°C for preservation. Cell samples were prepared for transcriptomic analysis of YN81 and CS31 treated with or without ethanol stress. The total RNA was extracted with a hot phenol method (Schmitt et al., 1990) followed by purification using RNeasy Mini Kit (Qiagen GmbH, Germany). mRNA library preparation was performed using a NEBNext mRNA Library Prep Reagent Set for Illumina according to the manufacturer’s instructions (New England Bio-Labs Inc., Ipswich, MA, United States). The cDNA libraries were sequenced using the Illumina HiSeq X10 platform (Illumina, San Diego, CA, United States). Adapters and low-quality reads were trimmed using FASTP version 0.19.5 (Bolger et al., 2014). Trimmed reads were mapped onto *S. cerevisiae* S288C reference genome (RefSeq assembly accession: GCF_000146045.2) using HISAT2 Version 2.1.0 (Mo et al., 2019). Gene expression levels were estimated using RSEM v.1.3.3 software (Li and Dewey, 2011) and recorded in reads per kilobase per million mapped reads (RPKM). Differential expression (DE) analysis was performed by using the DEseq2 (Wang et al., 2009). The genes with *p*-value ≤ 0.05 and Fold Change ≥ 2.0 were defined as differentially expressed. The differentially expressed genes (DEGs) were classified and enriched by Gene Ontology (GO) and KEGG Pathway annotation. Single nucleotide polymorphisms (SNPs) are genetic markers formed by single nucleotide variation in the genome. After excluding duplicated reads and reordering the bam alignment results of each sample, the GATK (V3.5) software was used to perform SNP calling (Chen et al., 2017).

### 50 L-scale beer brewing

50 L-scale beer brewing was performed with *S. cerevisiae* YN81 and CS31 in 15°P high-gravity wort with 5% (v/v) ethanol. The initial amount of inoculation for yeast cells are 1.5 × 10^7^ CFU/mL. The fermentations were carried out at 15°C for 14 days. Wort samples were withdrawn aseptically from the fermentor and placed directly on the ice every 24 h. Then, *S. cerevisiae* was harvested from the fermented wort by centrifugation (9,000 g, 10 min, 4°C) (Krogerus et al., 2018). The residual sugar content for all samples was analyzed by a 3, 5−dinitrosalicylic acid colorimetric method (Lei et al., 2012). The ethanol level (v/v) of samples was determined from the centrifuged and degassed fermentation samples using FermentoFlash Beer Analyzer (Funke-Dr. N. Gerber Labortechnik GmbH, Germany).

### Statistical analysis

All determinations were carried out in triplicate, and the experimental results obtained were expressed as means ± standard deviations (SD). Statistical calculation was performed by SPSS 24 (SPSS Inc., Chicago, IL, United States) for one-way ANOVA. Student–Duncan test was used for comparison of mean values among treatments, and to identify significant differences (*p* < 0.05) among treatments.

## Results and discussion

### Mutagenesis of *Saccharomyces cerevisiae* CS31

The lethality rates curves of UV and DES mutagenesis for CS31 strain are shown in Supplementary Figure 1. Based on a lethality rate of 70–80%, the optimal mutation time for UV mutagenesis of CS31 was determined as 120 s (Supplementary Figure 1A), and the optimal conditions for DES mutagenesis were 0.05%(v/v) (Supplementary Figure 1B) and 30 min (Supplementary Figure 1C).

The results of primary screening and rescreening of *S. cerevisiae* CS31 mutated by UV and DES are listed in Table 1. Eight mutants with a 10% larger colony diameter than control were primarily screened from YPD-ethanol screening medium, named as YN81, YN83, YN84, YN85, YN86, YN89, YN810, and YN811, respectively. Under ethanol stress, the cell viability of YN81, YN84, YN85, YN86, and YN810 was 95.89, 79.65, 81.85, 80.75, and 80.65%, respectively, which was 47.72, 22.66, 26.10, and 24.25% higher than that of CS31. The cell viability of YN81 was significantly higher than that of the other mutants (*p* < 0.05). UV-DES cooperative mutagenesis, a simple and effective optimization strategy, is widely used to obtain various higher production strains (Hedman et al., 2007). Therefore, in this study, YN81 was screened by UV-DES cooperative mutagenesis, and its cell viability under ethanol stress was 47.72% higher than that of the parental strain CS31 (Table 1).

**TABLE 1:** Primary screening and rescreening of mutant strains.

Strain	Colony diameter (mm)	Colony increase ratio (%)	Cell viability (%)	Cell viability increase ratio (%)
CS31	2.85 ± 0.04 f	0 g	64.95 ± 3.25 e	0 e
YN81	4.71 ± 0.08 b	65.10 ± 4.24 b	95.85 ± 1.95 a	47.72 ± 4.39 a
YN83	3.81 ± 0.06 d	33.55 ± 3.29 e	75.30 ± 2.10 c	16.02 ± 2.58 c
YN84	3.71 ± 0.03 d	30.14 ± 0.64 e	79.65 ± 3.45 b	22.66 ± 0.83 b
YN85	5.23 ± 0.06 a	83.44 ± 3.66 a	81.85 ± 2.55 b	26.10 ± 2.39 b
YN86	4.15 ± 0.05 c	43.57 ± 0.40 d	80.75 ± 1.55 b	24.45 ± 3.85 b
YN89	4.26 ± 0.09 c	49.20 ± 4.02 c	72.95 ± 2.55 cd	12.37 ± 1.70 cd
YN810	5.19 ± 0.04 a	81.90 ± 1.48 a	80.65 ± 2.56 b	24.25 ± 2.28 b
YN811	3.27 ± 0.07 e	14.50 ± 3.25 f	70.10 ± 1.90 d	8.01 ± 2.48 d

Primary screening: Colony diameter in YPD plates contained 9% (v/v) ethanol after UV-DES cooperative mutagenesis; rescreening of mutant strains: cell viability in YPD medium containing 9% (v/v) ethanol. Mean value ± SD from triplicate analysis; values with different letters (a, b, c, d, e,f, g) are significantly different at p < 0.05.

### Ethanol tolerance performance of YN81

**FIGURE 1:**
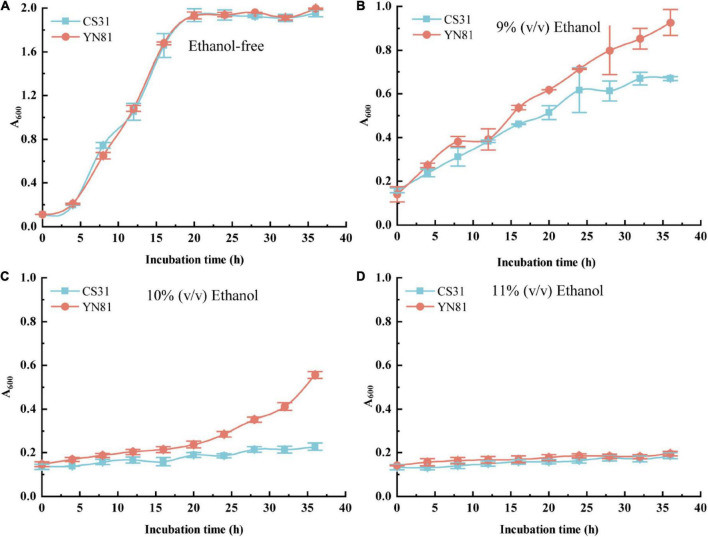
Cell growth curves of YN81 and CS31 under ethanol stress with different concentrations. the square filled with blue is CS31 and the circle filled with orange is YN81. **(A–D)** Cell growth curve of YN81 and CS31 in YPD medium containing 0, 9, 10, and 11% (v/v) ethanol, respectively.

To study the ethanol tolerance performance of mutant YN81, cell growth of YN81 at different concentrations of ethanol were investigated (Figure 1). With the increasing ethanol concentration, the growth rate of YN81 and CS31 cells gradually slowed down (Figure 1). The cell growth curves of YN81 and CS31 under ethanol-free stress were typical logarithmic curves, and there was no significant difference between the two strains (Figure 1A), but under 9% (v/v) ethanol stress, the cell growth of YN81 and CS31 showed a linear trend. While with the extension of incubation time, the growth rate of YN81 was significantly faster than that of CS31 (Figure 1B). The cell proliferation curve of CS31 was nearly horizontal under 10% ethanol stress, but for YN81 after 20 h, the cell growth curve of YN81 exhibited an increasing tendency. 36 h after culturing, the cell concentration of YN81 reached 0.669, which was 143.8% higher than that of CS31 (Figure 1C). Under the stress of 11% (v/v) ethanol, the cell growth curves of YN81 and CS31 were almost parallel to the x-axis, indicating that 11% (v/v) ethanol significantly inhibited their cell proliferation (Figure 1D). Meanwhile, the cell viability of YN81 and CS31 at various concentrations of ethanol was shown in Supplementary Figure 2. With the increasing ethanol concentration, the cell viability of YN81 and CS31 gradually decreased. There was no significant difference in the cell viability between YN81 and CS31 cells cultured without ethanol. Under 9% (v/v) ethanol stress, the cell viability of YN81 and CS31 cells was 90.72 and 66.83%, respectively. When YN81 and CS31 cells were exposed to 10% (v/v) ethanol, their cell viability was 60.18 and 24.66%, respectively. The result showed that the cell viability of YN81 was significantly greater than that of CS31. Whereas, under 11% (v/v) ethanol stress, there was no significant difference in cell viability between YN81 and CS31. As a result, the ethanol tolerance of YN81 was significantly higher than that of CS31.

The viability and proliferation of *S. cerevisiae* cells are closely related to the fermentation of high-gravity beer (Henderson et al., 2013; Brickwedde et al., 2017). With the increasing ethanol stress, the cell growth and cell viability of *S. cerevisiae* YN81 were inhibited inordinately (Figure 1 and Supplementary Figure 2). These results are similar to those reported by Birch and Walker (2000). Generally, cell viability under ethanol stress is related to many factors, such as cell membrane permeability and intracellular trehalose content (Petitjean et al., 2020).

Accordingly, the relationship of the high-ethanol tolerance of YN81 with the cell membrane permeability and trehalose content should be studied. Since 11% (v/v) ethanol significantly inhibited the cell growth of YN81 compared with 10% (v/v) ethanol, the ethanol tolerance mechanism of YN81 was studied subsequently under 10% (v/v) ethanol stress.

### Effect on cell morphology and membrane permeability under ethanol stress

**FIGURE 2:**
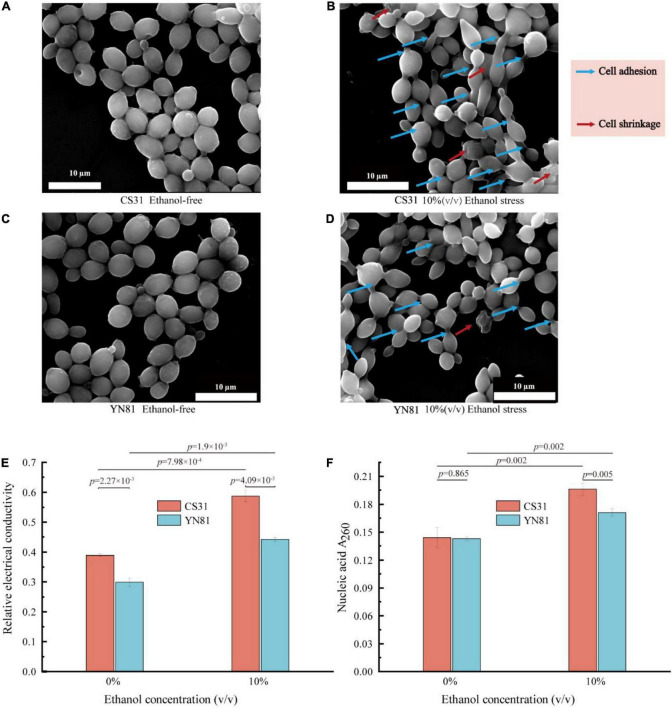
Effects of ethanol stress on *S. cerevisiae* morphology and membrane permeability. **(A,B)** SEM images of *S. cerevisiae* CS31 exposure to YPD media containing 0 and 10% (v/v) ethanol. **(C,D)** SEM images of *S. cerevisiae* YN81 exposure to YPD media containing 0 and 10% (v/v) ethanol. **(E)** REC of YN81 and CS31 cell membranes under 10% (v/v) ethanol stress. **(F)** YN81 and CS31 nucleic acid extravasation under 10% (v/v) ethanol stress.

YN81 and CS31 cells were cultivated in YPD medium containing 10% (v/v) ethanol to evaluate the effects on the cell morphology, and the results were presented in Figures 2A–D. In the ethanol-free medium, the cell morphology of the mutant YN81 and parental strain CS31 showed regular oval shapes with smooth surfaces and many bud scars, with no significant difference between them. However, under 10% (v/v) ethanol stress, YN81 and CS31 were found to have significant changes in cell morphology. Both *S. cerevisiae* YN81 and CS31 cells became smaller, the number of bud scars decreased, and cell adhesion and wrinkling appeared (Figures 2B,D), which agreed with the experimental results reported by Yang et al. (2018b). In fact, compared with CS31, mutant YN81 maintained better cell morphology under 10% (v/v) ethanol, while cell morphology of CS31 got worse. Therefore, when *S. cerevisiae* was subjected to high ethanol concentrations, the cell morphology was altered, mainly manifested as cell size, cell adhesion, and shrinkage (Udom et al., 2019).

On the other hand, changes in cell membrane permeability and the leakiness of nucleic acids in *S. cerevisiae* were detected. The permeability of cell membrane can be indirectly represented by the REC of the cell. The higher REC of the cell, the more damaged the integrity of the cell membrane will be (Saini et al., 2018). Under 10% ethanol stress, the REC of the mutant YN81 and parental strain CS31 were 0.442 and 0.587, increasing by 31.9 and 50.9%, respectively (Figure 2E). Additionally, under 10% ethanol stress, the amount of extracellular nucleic acids in YN81 and CS31 cultures increased by 19.6 and 36.1%, respectively (Figure 2F). The cell membrane permeability and leakage of nucleic acids indicated that high concentration ethanol would damage the integrity of the cell membrane, resulting in the exudation of intracellular substances.

The results of the experiment indicated that the high-ethanol tolerance of YN81 was closely related to cell membrane integrity (Zhao et al., 2014; Yang et al., 2018a). As a barrier between the cell and the external environment, the cell membrane plays a very important role in maintaining the stability of the intracellular environment and transport function (Lopez et al., 2021). When *S. cerevisiae* was exposed to high concentration ethanol, the lipids, a component of the cell membrane, and its long-chain hydrocarbyl were destroyed, resulting in a decline in the osmotic barrier function of the *S. cerevisiae* cell membrane, an increase in permeability, and the leakage of intracellular substances (Aguilera et al., 2006). However, *S. cerevisiae* could respond to ethanol stress by overexpressing membrane-related genes. For example, under high concentration ethanol, *S. cerevisiae* cells reduced the content of cell membrane lipids and then changed the fluidity of the cell membrane (Odumeru et al., 1993). Studies had shown that exogenous hexadecanoic acids and oleic acids could also change the composition of the cell membrane to improve tolerance to high concentration ethanol (You et al., 2003). *S. cerevisiae* could also improve tolerance to ethanol by synthesizing unsaturated fatty acids to change the cell membrane fluidity (Chen and Xu, 2014). Therefore, it was speculated that the ethanol tolerance ability of the mutant YN81 was higher than that of the parental strain CS31 and this phenomenon might be related to the cell membrane.

### Change of trehalose content and related genes under ethanol stress

**FIGURE 3:**
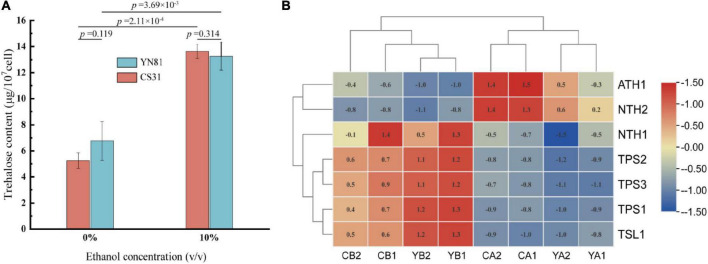
Trehalose content and trehalose-related genes expression in *S. cerevisiae* under ethanol stress. **(A)** Trehalose concentration of YN81 and CS31. **(B)** Expression of trehalose-related genes in YN81 and CS31; YA and YB represent the *S. cerevisiae* YN81 treated with ethanol-free and ethanol stress, respectively; CA and CB represent the *S. cerevisiae* CS31 treated with ethanol-free and ethanol stress, respectively.

Trehalose plays an important role in protecting cells to reduce stress-induced damage in the external environment (Chen et al., 2019). To study whether the high ethanol tolerance of the mutant YN81 than that of CS31 was related to intracellular trehalose content, the intracellular trehalose content was determined. The standard curve of trehalose content in *S. cerevisiae* was shown in Supplementary Figure 3 (y = 0.028–4.312x, *R*^2^ = 0.9991). The intracellular trehalose contents of YN81 and CS31 in YPD medium containing 10% ethanol were 13.26 and 13.64 μg/10^7^ cells, respectively, compared with ethanol-free medium (Figure 3A). Moreover, in the YPD medium containing 0 and 10% (v/v) ethanol, there were no significant differences in trehalose content between YN81 and CS31.

The expression of trehalose-related genes in YN81 was analyzed by cluster analysis based on the transcriptomic. The expression of the trehalase-related genes *ATH1* and *NTH2* in YN81 and CS31 were downregulated by ethanol stress, while that of the trehalose synthesis-related genes *TPS1*, *TPS2*, *TPS3*, and *TSL1* were upregulated (Figure 3B), which substantiated that the expression of trehalose-related genes was closely related to ethanol tolerance (Gibney et al., 2015). However, compared with that in the CS31 strain, there was no significant difference in trehalose-related genes expression in YN81, and therefore, the increase in ethanol tolerance of YN81 might be related to other pathways. The expression of trehalose-related genes could be regulated by ethanol stress in *S. cerevisiae*, resulting in a significant increase in trehalose content, which indicated that trehalose might be involved in ethanol stress (Malhotra and Singh, 2006), but there was no significant difference between ethanol tolerance and trehalose-associated genes for YN81 and CS31. Therefore, trehalose content was positively relevant to ethanol tolerance, and the expression of trehalose-related genes did not account for the ethanol tolerance ability of YN81 higher than CS31.

### Transcriptomic analyses of the mutant strain YN81

**FIGURE 4:**
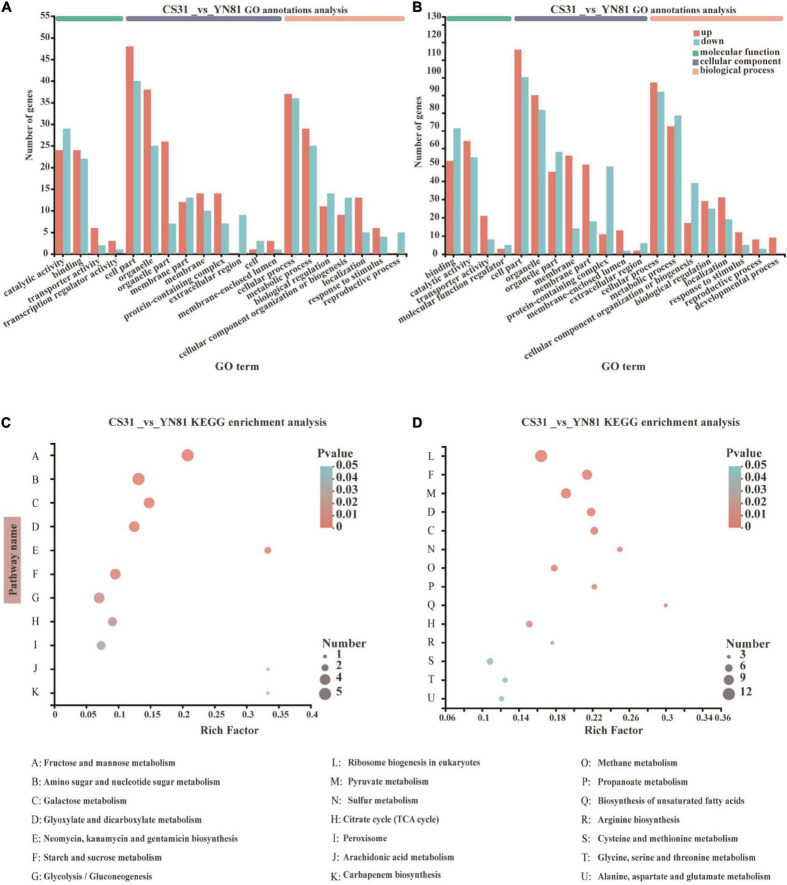
GO function annotation and KEGG enrichment analysis of the DEGs in *S. cerevisiae* CS31 and YN81. **(A,B)** Gene ontology (GO) terms of the DEGs in CS31 vs. YN81 at ethanol-free stress, and 10% (v/v) ethanol stress, respectively. The genes were assigned to three GO categories, biological process, cellular component, and molecular function. The horizontal axis indicated the top 20 ranked GO terms of DEGs. The vertical axis on the left represented the genes numbers annotated successfully by GO assignment. **(C,D)** Bubble diagram of top 20 ranked KEGG metabolism pathway of DEGs in CS31 vs. YN81 at ethanol-free stress, and 10% (v/v) ethanol stress, respectively. The vertical axis represented KEGG metabolism pathway and the horizontal axis indicated the Rich Factor. The enrichment degree was higher with a bigger Rich Factor. The sizes of dots manifested the number of genes in the KEGG metabolism pathway.

The expression of DEGs between YN81 and CS31 became more active under ethanol stress than ethanol-free stress (Figures 4A,B). Compared with CS31, there were 49 upregulated genes and 47 downregulated genes of YN81 under ethanol-free stress. GO function annotation analysis showed that these genes were mainly related to binding, catalytic activity, cell part, organelle part, cell process, and metabolic process. The expression of 130 genes and 114 genes were upregulated and downregulated by ethanol stress, respectively. Specifically, the number of upregulated genes in the cell membrane and cell membrane part was significantly higher than that of downregulated genes. Moreover, development process-related genes appeared in the biological process category, and it was speculated that the expression of genes related to catalytic molecules and development processes increased in mutant YN81 to adapt to ethanol stress.

In particular, the cell membrane and cell membrane parts might play a more important role under ethanol stress. Under ethanol-free stress, 26 upregulated genes and 20 downregulated genes associated with membrane and membrane part were found in the mutant YN81 (Figure 4A), while 103 upregulated genes and 32 downregulated genes were found under ethanol stress (Figure 4B). The results above were in agreement with Leach and Cowen’s (2014) report that the active expression of membrane-associated genes is closely related to the permeability and stability of the cell membrane. According to the GO functional annotation, it could be found that compared with CS31, more membrane-associated genes were expressed in YN81 under ethanol stress, which possibly improved the membrane stability of YN81.

The DEGs between mutant YN81 and parental strain CS31 with and without ethanol stress were enriched on the KEGG pathway. The results were shown in Figures 4C,D. Under ethanol-free stress, the pathways of KEGG enrichment (CS31 vs. YN81) were focused on fructose and mannose metabolism, amino sugar and nucleotide sugar metabolism, galactose metabolism, glyoxylate and dicarboxylate metabolism, neomycin and kanamycin metabolism, gentamicin metabolism, starch and sucrose metabolism, glycolysis, citrate cycle and peroxisome (Figure 4C). In addition to retaining glyoxylate acid and dicarboxylate acid metabolism, galactose metabolism, and the TCA cycle under ethanol stress, new metabolic pathways were found, such as eukaryotic ribosome biogenesis, starch and sucrose metabolism, pyruvate metabolism, sulfur metabolism, methane metabolism, propanoate metabolism, biosynthesis of unsaturated fatty acid, arginine biosynthesis, cysteine and methionine metabolism, glycine, serine and threonine metabolism, and alanine, aspartate and glutamate metabolism (Figure 4D), and the new metabolic pathways were mostly associated with glucose metabolism, energy metabolism, cell membrane composition, and amino acid metabolism, which is an agreement with a previous study (Li B. et al., 2020). To sum up, the ethanol tolerance of YN81 was closely related to the eukaryotic ribosome synthesis, starch and sucrose metabolism, pyruvate metabolism, biosynthesis of unsaturated fatty acids, and amino acid metabolism pathways.

Studies have found that when *S. cerevisiae* cells were exposed to ethanol stress, they can improve their tolerance to ethanol by changing the composition and structure of their cell membrane, such as increasing the proportion of unsaturated fatty acids in the cell membrane (Dong et al., 2015). Under ethanol stress, the expression level of cell membrane-associated genes of YN81 was higher than CS31, especially those associated with the synthesis of unsaturated fatty acids. The previous study on the role of unsaturated fatty acids under ethanol stress has indicated that unsaturated fatty acids could compensate for the decrease in membrane fluidity and improve the ethanol resistance of *S. cerevisiae* (Bradley et al., 2021). Herein, it was speculated that genes associated with the synthesis of unsaturated fatty acids in YN81 were involved in ethanol stress. Ethanol stress could also affect the function of ribosomes (Li et al., 2019) by changing the expression of ribosome biogenesis-associated genes, which was proven by the increase in the expression of genes related to the ribosome biogenesis pathway determined by KEGG enrichment analysis, but the relevant stress mechanism is not clear. In addition, according to the KEGG functional enrichment analysis of DEGs between YN81 and CS31, compared with ethanol-free stress, several newly added metabolic pathways were related to amino acid metabolism under ethanol stress. The KEGG enrichment analysis showed that the expression of amino acid metabolism-associated genes under ethanol stress became activated, responding to these stresses and changing the intracellular amino acid content, which was in accordance with those reported by Hirasawa et al. (2009), who indicated that overexpression of the genes for tryptophan biosynthesis (*Trp1-5*) increased ethanol stress tolerance. Furthermore, ethanol tolerance in *S. cerevisiae* could be enhanced by the exogenous addition of amino acids. Lei et al. (2013) supplemented the medium with lysine and histidine and found that both ethanol yield and ethanol stress tolerance ability increased dramatically. In a similar case, Yang et al. (2018a) indicated that the addition of a mixture of lysine and isoleucine was more effective in improving the resistance of *S. cerevisiae* to ethanol. Consequently, amino acids essentially could improve the ethanol tolerance ability of *S. cerevisiae*, and the ethanol tolerance ability of YN81 was higher than that of CS31, which might be related to amino acid metabolism.

Variant calling on the aligned transcriptomes of YN81 and CS31 under ethanol stress was performed by the SNPs analysis. 4063 unique SNPs were observed in the transcriptome of mutant strains (Supplementary Table 1), of which 53.62% were synonymous, 41.53 % were non-synonymous, and 4.26% were unknown. In addition, there were 283 genes involved in non-synonymous, and these genes are classified by KEGG pathways (Supplementary Table 2). The results showed there were 15 genes participating in amino acid-related metabolism and 7 genes involved in lipid metabolism, possibly related to the cell membrane. But the trehalose-related genes were not found. The results of SNPs analysis were similar to the above findings. Meanwhile, for the non-sense mutation, 21 stop gain and 3 stop loss genes were studied and annotated in detail (Supplementary Table 3). Among 24 non-sense mutations, 13 were homozygous mutations and 11 heterozygous mutations. Furthermore, we found that the three genes, *MET4* (Mardanov et al., 2020), *HXT17* (Li et al., 2019), and *HSP32* (Ma and Liu, 2010), had been reported in previous studies, might be related to ethanol tolerance.

### Mechanism analysis on ethanol tolerance of YN81

**FIGURE 5:**
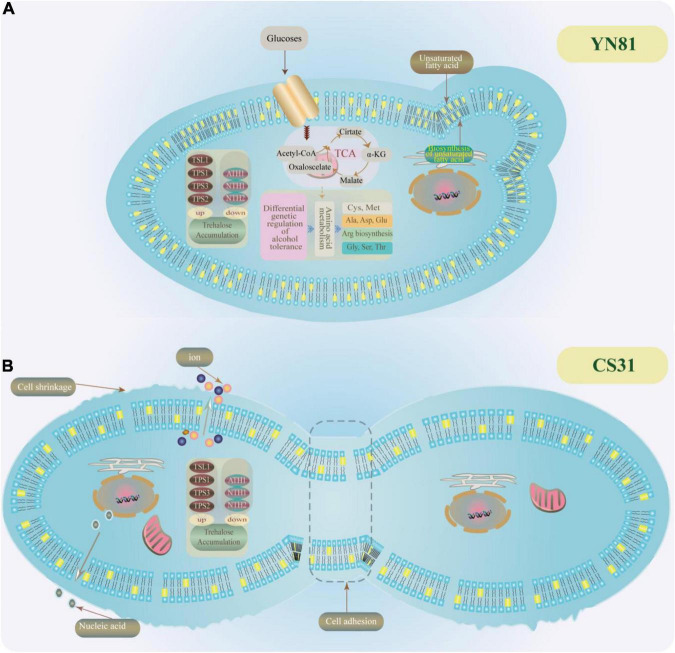
Possible mechanisms for ethanol tolerance in YN81. **(A,B)** YN81 and CS31 under 10% (v/v) ethanol stress.

To demonstrate the intrinsic causes that YN81 had a higher ability in ethanol tolerance than CS31, we drew a diagram of underlying mechanisms on ethanol tolerance of YN81, which was shown in Figure 5. Based on our research results and previous reports, we speculated that there may be several reasons for the high ethanol tolerance of YN81 as follows. (1) The cell membrane of YN81 was more stable than that of CS31, which was crucial for *S. cerevisiae* to be resistant to ethanol. (2) The equilibrium of cell membrane permeability could reduce the leakage of intracellular ions and nucleic acids in YN81. (3) Due to the active expression of trehalose-related genes, trehalose content was accumulated in the intracellular to increase ethanol tolerance of *S. cerevisiae*, however, the accumulation of trehalose content was not the reason for the high ethanol tolerance of YN81. (4) The genes related to the synthesis of unsaturated fatty acids in YN81 were involved in high ethanol tolerance. (5) Amino acids metabolic pathway plays a critical role in high ethanol tolerance mechanism of YN81. However, the intrinsic mechanisms why the ethanol tolerance of YN81 was higher than CS31 still need intensive investigation.

### The high-gravity beer brewing with YN81

YN81 was used for high-gravity beer in the 50 L-scale cylinder conical tank. Fermentation experiments were conducted using 15^°^P all-malt wort containing 5% (v/v) ethanol. The residual reducing sugar and ethanol production during fermentation was shown in Supplementary Figure 4. Reducing sugar in the wort was continuously decreased as the fermentation progressed. After 336 h of fermentation, the residual reducing sugar content of YN81 and CS31 was the lowest, at 6.11 and 25.39 g/L, respectively, which showed the reducing sugar utilization ability of YN81 was significantly higher than that of CS31. Simultaneously, we also measured alterations of ethanol production during fermentation (Supplementary Figure 4). After 96 h, the ethanol yield of YN81 began to be higher than that of CS31, reaching 10.16% (v/v) at 312 h, while the ethanol yield of CS31 was only 8.62% (v/v). YN81 was more efficient in reducing sugar utilization than CS31 in 15°P all-malt wort with 5% (v/v) ethanol (Supplementary Figure 4). The residual sugar content of CS31 was 3 times that of YN81 at the end of fermentation. Furthermore, the ethanol tolerance ability of YN81 was significantly higher than that of CS31, and the ultimate ethanol concentration was 17.86% higher than that of CS31. As a result, in the all-malt wort fermentation test, the fermentation capacity and alcohol production capacity of YN81 were greater than that of CS31.

## Conclusion

In summary, to address the issue of ethanol stress for *S. cerevisiae* in high-gravity brewing, obtaining high ethanol tolerance *S. cerevisiae* and elucidating its tolerance mechanism is critical. In this study, we screened a mutant YN81 with high ethanol tolerance from CS31, and revealed that trehalose was associated with ethanol tolerance for both YN81 and CS31, while the high ethanol tolerance of YN81 had nothing to do with intracellular trehalose content, compared with CS31. Furthermore, the high ethanol tolerance of YN81 was inextricably related to the cell membrane and amino acid metabolic pathways. This research provides an advantageous strain material as well as a solid theoretical foundation for high-gravity brewing.

## Data availability statement

The datasets presented in this study can be found in online repositories. The names of the repository/repositories and accession number(s) can be found below: https://www.ncbi.nlm.nih.gov/bioproject?term=PRJNA855342&cmd=DetailsSearch, NCBI, PRJNA855342.

## Author contributions

TY, SZ, LL, JT, XL, and YP designed and performed research study. TY, SZ, and LL wrote the manuscript. All authors contributed to the article and approved the submitted version.
