# Supplementary material for: Screening and transcriptomic analysis of the ethanol-tolerant mutant Saccharomyces cerevisiae YN81 for high-gravity brewing
Source: Front Microbiol. 2022 Aug 25;13:976321. doi: 10.3389/fmicb.2022.976321 (PMC9453260; doi:10.3389/fmicb.2022.976321)
Supplement: Supplementary file 1 [file Data_Sheet_1.pdf]

Supplementary Material

Supplementary Figures

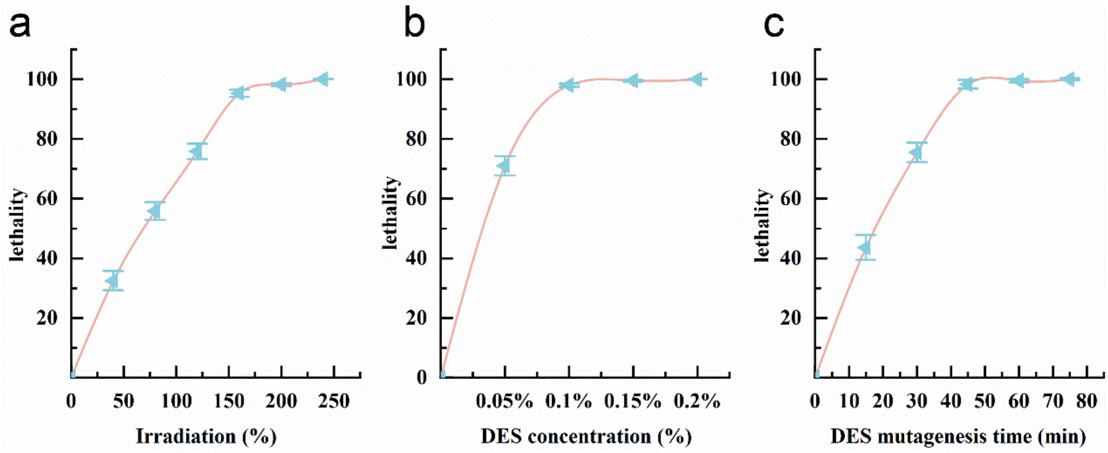

Supplementary Figure 1. Determination of UV-DES cooperative mutagenesis conditions. a: lethality of CS31 in different UV irradiation times. b: lethality of CS31 in 30 min of DES mutagenesis at different concentrations. c: lethality of CS31 in different times of DES mutagenesis for 0.05% (v/v).

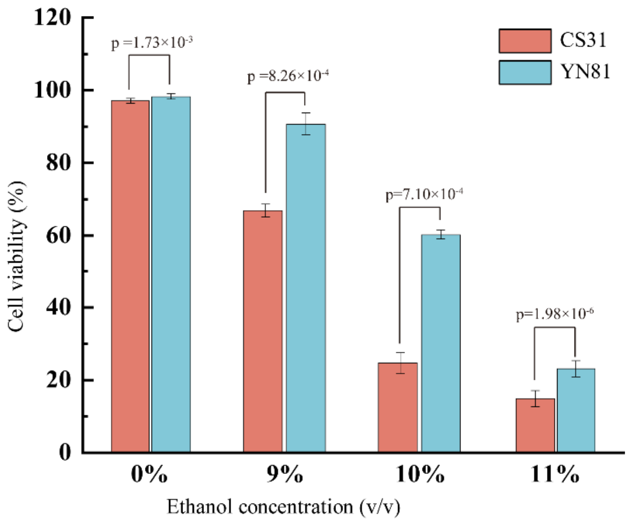

Supplementary Figure 2. Cell viability of *Saccharomyces cerevisiae* under ethanol stress with different concentration. Cell viability of YN81 cultured in YPD medium at different ethanol concentrations (0, 9, 10, and 11% (v/v)) for 36h, orange represents CS31, blue filled is YN81.

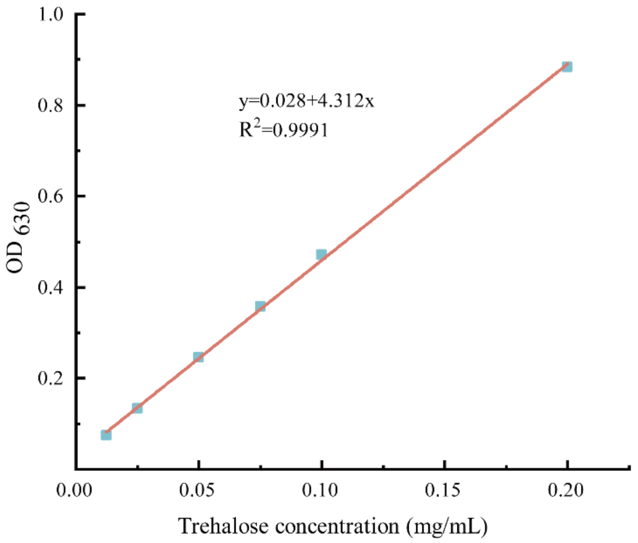

Supplementary Figure 3. Standard curve of trehalose concentration. The absorbance values of different standard concentrations of trehalose at wavelength 630nm and fitted linear curve (y=0.028+4.312x, R_2=0.9991).

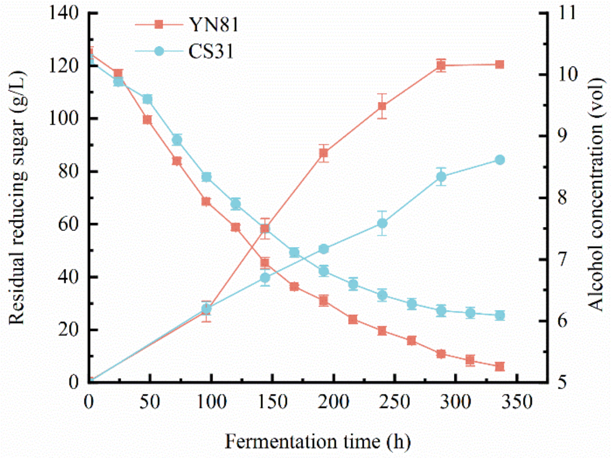

Supplementary Figure 4. The high-gravity beer brewing by YN81. YN81 and CS31 were fermented in all-malt wort (15°P) containing 5% (v/v) ethanol at 15°C for 14 days, the triangle filled with orange represents YN81 and the circle filled with blue represents CS31.

Supplementary Tables

Supplementary Table 1. SNPs and Indel analysis of strains YN81 and CS31 under ethanol stress

Mutation type	SNPs number	SNPs percent
Non-synonymous	1687	41.53%
Stop gain	21	0.52%
Stop loss	3	0.07%
Synonymous	2178	53.62%
Frameshift mutations	0	0
Unknown	173	4.26%
Indel	0	0
Total	4063	100%

Supplementary Table 2. Classification of non-synonymous genes

Gene Pathway	Gene number
Cellular Processes	
Cell growth and death	74
Transport and catabolism	24
Environmental Information Processing	
Membrane transport	3
Signal transduction	31
Genetic Information Processing	
Folding, sorting and degradation	17
Replication and repair	16
Transcription	5
Translation	26
Metabolism	
Amino acid metabolism	11
Biosynthesis of other secondary metabolites	1
Carbohydrate metabolism	31
Energy metabolism	6
Glycan biosynthesis and metabolism	4
Lipid metabolism	7
Metabolism of cofactors and vitamins	10
Metabolism of other amino acids	4
Metabolism of terpenoids and polyketides	1
Nucleotide metabolism	6
Xenobiotics biodegradation and metabolism	1
Organismal Systems	
Aging	4

Supplementary Table 3 Annotation of stop loss and stop gain genes

Mutation type	Gene name	Gene description	Chromosome	Position	Mutation information	Genotype
stop loss	HMRA2	homeodomain mating type protein a2	NC_001135.5	293181	gene-YCR096C:rna-NM_001178802.1: exon1:c.T358C:p.X120R,	A/G
	YNR066C	uncharacterized protein	NC_001146.8	753727	gene-YNR066C:rna-NM_001183243.3: exon1:c.T1309G:p.X437E gene-YNR066C:rna-NM_001183243.3: exon1:c.T1309A:p.X437K	C/C
	KAP120	Kap120p	NC_001148.4	316484	gene-YPL125W:rna-NM_001183939.1: exon1:c.T3097C:p.X1033R,	T/T
stop gain	YPR202W	uncharacterized protein	NC_001148.4	943858	gene-YPR202W:rna-NM_001184299.1: exon2:c.C679T:p.Q227X,	C/T
	**MET4**	Met4p	NC_001146.8	428386	gene-YNL103W:rna-NM_001182941.3: exon1:c.C652T:p.R218X,	C/T
	YLR410W-A YLR410W-B	gag protein;;gag-pol fusion protein	NC_001144.5	941570	gene-YLR410W-A:rna-NM_001184410.1: exon1:c.C88T:p.Q30X,	C/T
	ASK1	Ask1p	NC_001143.9	339934	gene-YKL052C:rna-NM_001179618.1: exon1:c.C613T:p.Q205X,	G/A
	**HXT17**	hexose transporter HXT17	NC_001146.8	773443	gene-YNR072W:rna-NM_001183249.1: exon1:c.G787T:p.E263X,	G/T
	VIP1	inositol polyphosphate kinase VIP1	NC_001144.5	939475	gene-YLR410W:rna-NM_001182298.1: exon1:c.G1935A:p.W645X,	G/A
	CLN1	cyclin CLN1	NC_001145.3	664241	gene-YMR199W:rna-NM_001182706.1: exon1:c.C1598A:p.S533X,	C/A

Continue Supplementary Table 3 Annotation of stop loss and stop gain genes

Mutation type	Gene name	Gene description	Chromosome	Position	Mutation information	Genotype
	HDA3	Hda3p	NC_001148.4	893814	gene-YPR179C:rna-NM_001184276.1: exon1:c.A1951T:p.K651X,	T/A
	THI22	putative phosphomethylpyrimidine kinase	NC_001148.4	780158	gene-YPR121W:rna-NM_001184218.2: exon1:c.C1576T:p.Q526X,	C/T
	TOP1	DNA topoisomerase 1	NC_001147.6	315126	gene-YOL006C:rna-NM_001183260.1: exon1:c.A262T:p.K88X,	T/A
	PMD1	Pmd1p	NC_001137.3	426332	gene-YER132C:rna-NM_001179022.3: exon1:c.T4118A:p.L1373X,	T/T
	YOR389W	uncharacterized protein	NC_001147.6	1075230	gene-YOR389W:rna-NM_001183809.3: exon1:c.C1018T:p.Q340X,	C/C
stop gain	TOG1	Tog1p	NC_001137.3	556448	gene-YER184C:rna-NM_001179074.1: exon1:c.G2233T:p.E745X,	A/A
	ECM1	Ecm1p	NC_001133.9	36524	gene-YAL059W:rna-NM_001178201.2: exon1:c.C16T:p.R6X,	T/T
	YFL015C	uncharacterized protein	NC_001138.5	106510	gene-YFL015C:rna-NM_001348832.1: exon1:c.G454T:p.E152X,	A/A
	YFL015C	uncharacterized protein	NC_001138.5	106482	gene-YFL015C:rna-NM_001348832.1: exon1:c.G482A:p.W161X,	C/C
	MMO1	Mmo1p	NC_001143.9	356466	gene-YKL044W:rna-NM_001270755.1: exon1:c.C145T:p.Q49X,	C/C
	YFL064C	uncharacterized protein	NC_001138.5	3360	gene-YFL064C:rna-NM_001179903.1: exon1:c.C487T:p.Q163X,	A/A

Continue Supplementary Table 3 Annotation of stop loss and stop gain genes

Mutation type	Gene name	Gene description	Chromosome	Position	Mutation information	Genotype
	**HSP32**	glutathione-independent methylglyoxalase family protein	NC_001148.4	12054	gene-YPL280W:rna-NM_001184094.1: exon1:c.G168A:p.W56X,	G/G
	YLL067C	Y' element ATP-dependent helicase	NC_001144.5	3893	gene-YLL067C:rna-NM_001181887.1: exon2:c.C310T:p.R104X,	G/G
	ATF1	alcohol O-acetyltransferase	NC_001147.6	1046771	gene-YOR377W:rna-NM_001183797.3: exon1:c.G546A:p.W182X,	G/G

Note: Genes that may be associated with high ethanol tolerance of YN81 are in bold; the difference before and after “/” is heterozygous, and the same before and after “/” is homozygous
